# Sarcoïdose systémique révélée par une hypertension intracrânienne

**DOI:** 10.11604/pamj.2015.20.307.6444

**Published:** 2015-03-30

**Authors:** Madiha Mahfoudhi, Sami Turki

**Affiliations:** 1 Service de Médecine Interne A, Hôpital Charles Nicolle, Tunis, Tunisie

**Keywords:** Sarcoïdose, hypertension intracrânienne, granulome, Sarcoidosis, intracranial hypertension, granuloma

## Image en medicine

La sarcoïdose systémique est une maladie ganulomateuse qui touche avec prédilection le poumon et le médiastin. Elle peut s'accompagner de complications graves particulièrement neurologiques. L'hypertension intracrânienne est rarement révélatrice de cette pathologie. Patiente âgée de 35 ans a consulté pour des céphalées, des vomissements, une xérostomie et des troubles de la marche. L'examen physique a objectivé un syndrome cérébelleux. L'examen biologique a retrouvé un syndrome inflammatoire, un taux élevé de l'enzyme de conversion de l'angiotensine, une calcémie et une calciurie normales, un bila rénal et hépatique sans anomalies. La tomodensitométrie cérébrale a révélé une hydrocéphalie avec dilatation du ventricule latéral gauche. L'IRM cérébrale a retrouvé cette dilatation associée à la présence d'un granulome ayant un aspect d'hypersignal en T2 et obstruant partiellement le trou de Monro gauche. L’étude du liquide céphalo-rachidien a montré une hyperprotéinorrachie avec une cytologie normale. Les diagnostics de tuberculose, sarcoïdose et lymphome ont été suspectés. L'examen anatomopathologique des biopsies labiales et trans-bronchiques étagées a conclut à la présence de granulomes épithéloïdes et giganto-cellulaires sans nécrose caséeuse. La recherche de bacille acido-alcoolo-résistant par la coloration de Ziehl-Neelson était négative. La culture sur milieu de Lobstein ainsi que l'intradermo-réaction à la tuberculine étaient aussi négatives. Le diagnostic de sarcoïdose systémique compliquée d'une atteinte neurologique a été retenu. Par ailleurs les explorations fonctionnelles respiratoires, le scanner thoracique et le lavage alvéolaire étaient sans anomalies. Le traitement s'est basé sur une corticothérapie associée à des boli de cyclophosphamide. L’évolution était favorable sur le plan clinique et radiologique.

**Figure 1:**
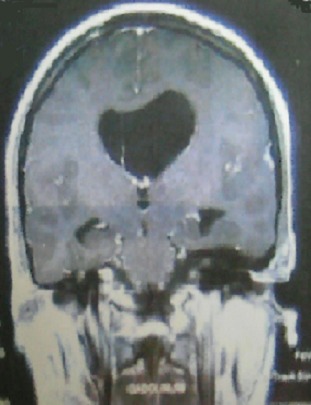
TDM cérébrale (coupe coronale): dilatation du ventricule latéral gauche

